# Cardiovascular events and mortality in adults with late onset seizures: Focus on enzyme‐modulating antiseizure medications

**DOI:** 10.1002/epi.70389

**Published:** 2026-07-18

**Authors:** Paola Vassallo, Nathan A. Shlobin, Mark R. Keezer, Roland D. Thijs, Ley Sander

**Affiliations:** ^1^ UCL Queen Square Institute of Neurology London UK; ^2^ Chalfont Centre for Epilepsy UK; ^3^ Stichting Epilepsie Instellingen Nederland Heemstede the Netherlands; ^4^ Department of Neurology Leiden University Medical Center Leiden the Netherlands; ^5^ Department of Neurosurgery, Neurological Institute of New York New York Presbyterian Hospital–Columbia University Medical Center New York New York USA; ^6^ Neurology Division Centre Hospitalier de L'université de Montréal Montreal Quebec Canada; ^7^ Centre de Recherche Centre Hospitalier de L'université de Montréal Montreal Quebec Canada; ^8^ School of Public Health Université de Montréal Montreal Quebec Canada; ^9^ Department of Neurology, West China Hospital Sichuan University Chengdu China

**Keywords:** adverse events, comorbidities, late onset epilepsy

## Abstract

**Objective:**

Using a Dutch insurance claims registry, we examine whether people with epilepsy have an increased risk of major ischemic cardiovascular events or death compared to people without epilepsy. We also assess whether this risk differs between users of enzyme‐modulating and non‐enzyme‐modulating antiseizure medications (ASMs).

**Methods:**

This nationwide study included adults ≥50 years old in the VEKTIS registry. Incident seizure cases were matched 2:1 to controls by age, sex, and region. ASM prescriptions (≥6 months) were classified as enzyme‐modulating or non‐enzyme‐modulating. The primary outcome was a composite event over 5‐year follow‐up. Cox models adjusted for age, sex, and baseline cardiovascular medications.

**Results:**

We matched 8728 cases to 17 445 controls (56% male, median age = 67 years, range = 50–102). During follow‐up, 6895 events occurred. Seizure disorder was associated with a higher cardiovascular risk than controls (hazard ratio [HR] = 2.30, 95% confidence interval [CI] = 2.19–2.41, *p* < .001), particularly early after diagnosis. Older age and cardiovascular medication use increased risk, whereas female sex was protective. Among 1758 participants with seizures treated with ASM, 656 events occurred. The enzyme‐modulating ASM group showed a nonsignificant trend toward higher risk than the non‐enzyme‐modulating ASM group (HR = 1.14, 95% CI = .97–1.34, *p* = .12). Time‐varying models suggested a lower risk with current enzyme‐modulating ASM exposure compared with non‐enzyme‐modulating ASMs (HR = .80, 95% CI = .70–.92, *p* = .001). There was an increased risk with cumulative exposure (HR = 1.41 per year of past enzyme‐modulating ASM exposure, 95% CI = 1.30–1.54, *p* < .001).

**Significance:**

People with seizures face higher cardiovascular risk, especially shortly after diagnosis. Although short‐term analyses showed no clear differences between ASM categories, longer term and cumulative exposure to enzyme‐modulating ASMs was associated with modestly increased risk. Findings are generalizable to routine care, although further work is needed to clarify time‐ and drug‐specific cardiovascular effects.

**Key points**

- People with seizures have significantly higher risk of cardiovascular events or death compared with matched controls.
- In individuals with a seizure diagnosis, the risk is highest shortly after diagnosis.
- Short‐term enzyme‐modulating ASM use was not significantly associated with the composite outcome during the limited follow‐up.
- Sustained or cumulative exposure to enzyme‐modulating ASMs may be associated with increased risk of the composite outcome.

## 1 INTRODUCTION

People with epilepsy experience higher rates of cardiovascular comorbidities and cardiovascular‐related death compared to the general population.^1^ The concept of the “epileptic heart” describes how chronic epilepsy may induce structural and functional cardiac changes.^2^ Certain antiseizure medications (ASMs) have also been implicated in adverse cardiac effects, including arrhythmias, atherosclerosis, and valvulopathies.^3^ These risks coexist with other contributing factors such as lifestyle, comorbidities, and socioeconomic disparities.^4^

Enzyme‐inducing ASMs (EIASMs), including phenytoin, phenobarbital, and carbamazepine, increase the activity of enzymes involved in cholesterol synthesis, resulting in elevated levels of total and low‐density lipoprotein cholesterol.^5^, ^6^

Conversely, sodium valproate, an enzyme inhibitor, does not cause such metabolic changes. However, its overall vascular effects are uncertain, as it may induce atherosclerosis through mechanisms related to insulin resistance, weight gain, metabolic syndrome, and increased oxidative stress.^1^ Overall, the cardiovascular risk attributable to enzyme‐modulating ASMs (EMASMs) remains unclear.

Using an insurance claims registry, we examined whether people with epilepsy are at increased risk of major ischemic cardiovascular events or death compared with people without epilepsy. We also assessed whether this risk differs between users of EMASMs and non‐enzyme‐modulating ASMs (NEMASMs).

Our first question is whether people with seizures are more likely to experience a major ischemic cardiovascular event or death following diagnosis, with the hypothesis that risk will be higher than in controls. The second question focuses on assessing whether EMASM use in people with epilepsy is associated with increased risk compared to NEMASMs.

## 2 MATERIALS AND METHODS

### 2.1 Database

We used the VEKTIS national insurance claims registry, which covers 99.9% of the Dutch population under mandatory health insurance. Individuals are followed longitudinally through encrypted identifiers. Specialist care is recorded using Diagnosis–Treatment Combination (DTC) codes, which include the speciality, diagnosis (International Classification of Diseases, 10th Revision [ICD‐10]), and treatments delivered. Medication data are derived from pharmacy dispensing records and include Anatomical Therapeutic Chemical codes, prescription dates, and defined daily doses.^7^ Demographics (sex, birth year, death date, and truncated postal code), seizure disorder diagnosis dates, and cardiovascular diagnoses (myocardial infarction, transient ischemic attack, ischemic stroke) with department and event dates were available.

### 2.2 Case ascertainment and study population

To capture incident cases, we selected 2014 as the index year, with a 2‐year washout period (2012–2013) and a 5‐year follow‐up period (2015–2019). Cases were individuals ≥50 years old with a seizure disorder DTC code (Neurology 0330: 0601/0602) in 2014, with or without ASM prescriptions. Controls were individuals ≥50 years old without epilepsy‐related DTC codes between 2012 and 2019, matched 2:1 to cases by sex, birth year, and the first two postal‐code digits.

### 2.3 Exposure and outcome definitions

Exposure was classified according to the first ASM prescribed after the seizure disorder diagnosis, comparing EMASMs versus NEMASMs.^8^ Only prescriptions lasting more than 6 months after diagnosis were considered. Individuals with multiple first ASM prescriptions were excluded to avoid exposure misclassification, particularly when initial prescriptions spanned different ASM categories. EMASMs included carbamazepine, phenytoin, phenobarbital, primidone, oxcarbazepine, topiramate, and valproic acid; NEMASMs included lamotrigine, levetiracetam, lacosamide, zonisamide, pregabalin, and gabapentin.^9^

Comorbidities and cardiovascular risk factors are not directly coded in the VEKTIS database. Thus, baseline cardiovascular medication use before study enrollment was included in all models as a proxy for underlying cardiovascular disease burden.

Baseline cardiovascular medications (pre‐ASM initiation or before study enrollment) were grouped into antihypertensives/antiarrhythmics, antithrombotics/anticoagulants, lipid‐lowering drugs, and antidiabetics.

The composite outcome was incident ischemic cardiovascular disease (myocardial infarction, transient ischemic attack (TIA), ischemic stroke) or death. To maximize diagnostic reliability, cardiovascular events were restricted to cardiology (non‐ST‐elevation myocardial infarction/ST‐elevation myocardial infarction) or neurology (TIA/ischemic stroke) departments. Death was included regardless of cause. Participants were followed until the first event or censoring. Clinical details (syndrome classification and epilepsy severity, comorbidities, risk factors, and investigation results) were unavailable.

### 2.4 Statistical analyses

#### 2.4.1 Cox regression

For question 1, Cox proportional hazards models estimated hazard ratios (HRs) for the composite outcome, adjusting for case/control status, sex, age, and baseline cardiovascular medication use (none, one, or ≥2 classes) before enrollment. For question 2, we used Cox proportional hazards regression to compare individuals initiating EMASMs versus NEMASMs. Six‐month prescription intervals for the first ASM were considered to ensure biological relevance. Only events that occurred after seizure diagnosis and after ASM introduction were included. Models adjusted for ASM type and baseline cardiovascular medication use before ASM introduction (yes/no). Age and sex were included in all models despite matching to account for residual imbalance and improve the precision of effect estimates. Proportional hazards violations were addressed via stratification or time‐varying covariates. Multicollinearity was assessed before modeling. Treatment was classified as active if a prescription was recorded within 30 days before the event; otherwise, it was considered inactive. In the sensitivity analyses, we repeated the Cox proportional hazards regression to compare individuals who started EMASMs with those who started NEMASMs, irrespective of indication.

We also analyzed a subgroup of adults with seizure disorders prescribed ASM treatment for at least 6 months who remained within the same exposure category despite switching compounds. We also conducted a subgroup analysis in which current ASM exposure (NEMASM vs. EMASM, including in combination therapy) was modeled as a binary time‐varying covariate, adjusting for prior exposure to EMASMs, age, sex, and baseline cardiovascular drug use.

Cumulative EMASM exposure was defined as the sum of time spent on EMASM over the follow‐up period and modeled as a continuous, time‐varying variable (in years), with a 1‐interval lag to ensure temporal ordering. In time‐varying models, an individual may contribute to multiple exposure periods but can experience at most one event. Analyses were conducted in RStudio (Posit Software).

### 2.5 Ethics and privacy

Data were anonymized and accessible only to VEKTIS personnel within a secure EU‐based workspace. The study was exempt from ethics review, because it used deidentified administrative data.

## 3 RESULTS

### 3.1 Study summary

Eight thousand seven hundred twenty‐eight participants with a seizure disorder code were matched to 17 445 controls without a seizure code. Fifty‐six percent of participants were male, with a median age of 67 years (range = 50–102). Demographic features, events, ASM prescriptions, and cardiovascular drug coprescriptions at baseline are detailed in Table 1.

**TABLE 1:** Demographic features of the study population.

Variable	Total of all subjects, *N* = 26 173	Controls, *n* = 17 445	Seizure disorder, *n* = 8728	Total of seizure disorder treated with ASM, *n* = 1758	Non‐enzyme‐modulating ASM, *n* = 1147	Enzyme‐modulating, ASM *n* = 611
Median age, years (range)	67 (50–102)	67 (50–102)	67 (50–102)	69 (50–102)	70 (50–102)	68 (50–99)
Male, *n* (%)	14 772 (56.4%)	9846 (56.4%)	4926 (56.4%)	1061 (60.4%)	677 (59.3%)	384 (62.3%)
Cardiovascular drugs at baseline, *n* (%)						
None	11 191 (42.8%)	7907 (45.3%)	3284 (37.6%)	366 (20.8%)	221 (19.4%)	145 (23.5%)
One	5073 (19.4%)	3404 (19.5%)	1669 (19.1%)	319 (18.1%)	198 (17.3%)	121 (19.6%)
Two or more	9909 (37.9%)	6134 (35.2%)	3775 (43.3%)	1073 (61%)	723 (63.3%)	350 (56.8%)
Composite event, *n* (%)						
None	18 890 (72.2%)	13 732 (78.7%)	5158 (59.1%)	1102 (62.7%)	725 (63%)	377 (62%)
Any	7283 (27.8%)	3713 (21.3%)	3570 (40.9%)	656 (37.3%)	422 (36.9%)	234 (38%)
NSTEMI	373 (1.4%)	253 (1.5%)	120 (1.4%)	542 (30.8%)	13 (1.1%)	3 (.5%)
STEMI	253 (1%)	178 (1%)	75 (.9%)	16 (.9%)	5 (.4%)	1 (.2%)
TIA	622 (2.4%)	333 (1.9%)	289 (3.3%)	6 (.3%)	28 (2.5%)	12 (1.9%)
Stroke	1174 (4.5%)	561 (3.2%)	613 (7%)	40 (2.3%)	33 (2.9%)	19 (3.1%)
Death	4861 (18.6%)	2388 (13.7%)	2473 (28.3%)	52 (3%)	343 (29.9%)	199 (32.6%)

*Note*: Reported are the number of participants in the control and seizure disorder groups and subgroup with ASM treatment (non‐enzyme‐modulating vs. enzyme‐modulating); proportion of males; and number of cardiovascular medications prescribed before study enrollment (antihypertensive, antidiabetic, lipid‐lowering, antithrombotic, or anticoagulant agents). Numbers and proportions of outcomes are reported for NSTEMI, STEMI, TIA, ischemic stroke, death, none, or any event. Abbreviations: ASM, antiseizure medication; NSTEMI, non‐ST‐elevation myocardial infarction; STEMI, ST‐elevation myocardial infarction; TIA, transient ischemic attack.

Seven thousand five hundred forty‐nine participants received at least one ASM prescription. The first prescribed ASMs in controls were most frequently pregabalin, gabapentin, and clonazepam, accounting for 80% of the total ASM prescriptions. Levetiracetam, valproic acid, and carbamazepine were the most frequent first prescriptions in people with seizure code. The proportions of ASM prescriptions in epilepsy and in controls are shown in Figure 1A,B.

**FIGURE 1:**
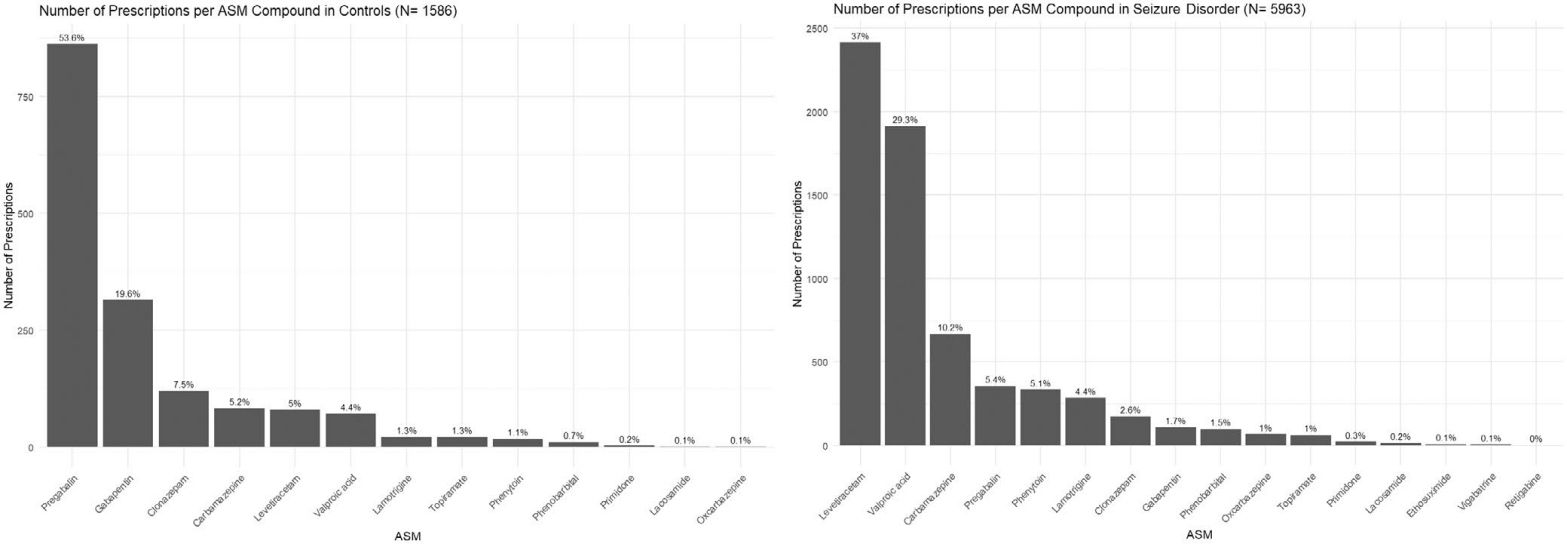
Number and proportion of first antiseizure medication (ASM) prescriptions in participants with seizure disorder versus controls. First ASM prescriptions among participants with a seizure diagnosis code are shown, with proportion of each ASM among all prescriptions in this group in controls (left panel) and seizure disorders (right panel).

Overall, ASM prescription preceded the seizure diagnosis recorded date, with a median time ranging from 168 to 525 days, except for the most prescribed ASMs, levetiracetam and valproic acid, which were mainly prescribed at the time of seizure diagnosis. Delays in ASM prescriptions in people with seizure disorders are displayed in Table S1.

### 3.2 Risk of composite outcome

To assess the risk of a composite outcome in people with seizure disorder versus controls, we included 26 173 participants in the Cox proportional hazards analysis. Six thousand eight hundred ninety‐five events were observed during follow‐up.

In the initial Cox proportional hazards model, individuals with seizure disorder had a significantly higher risk of the composite outcome compared with controls (HR = 2.30, 95% confidence interval [CI] = 2.19–2.41, *p* < .001). The model included age, sex, and cardiovascular treatment prescribed before study enrollment. Increasing age was associated with a higher hazard of the composite outcome (HR per year = 1.07, 95% CI = 1.07–1.07, *p* < .001), whereas female sex was associated with a significantly lower hazard compared with males (HR = .79, 95% CI = .75–.83, *p* < .001). Prescription of more than two cardiovascular drugs at baseline was associated with an increased hazard (HR = 1.19, 95% CI = 1.12–1.26, *p* < .001), whereas prescription of a single cardiovascular drug was not significantly associated with the outcome (HR = 1.02, 95% CI = .95–1.10, *p* = .56).

Assessment of the proportional hazards assumption identified violations for age and population group. To account for nonproportionality, a stratified Cox model with time‐dependent effects was fitted. In this model, the seizure disorder group remained at significantly higher risk of the composite outcome compared with controls, with the strongest effect observed in the early follow‐up period (first 540 days after seizure disorder diagnosis; HR = 3.69, 95% CI = 3.40–3.99, *p* < .001 in stratum 1; HR = 1.72, 95% CI = 1.61–1.83, *p* < .001 in stratum 2). Age was positively associated with the hazard in both time strata (HR per year = 1.05, 95% CI = 1.04–1.05 in stratum 1; HR = 1.08, 95% CI = 1.08–1.08 in stratum 2; both *p* < .001). Female sex remained associated with a lower hazard (HR = .79, 95% CI = .75–.83, *p* < .001). Baseline cardiovascular drug use was associated with increasing risk, with no significant effect for one drug (HR = 1.02, 95% CI = .95–1.09, *p* = .6) and a significantly higher hazard for participants taking more than two drugs (HR = 1.19, 95% CI = .95–1.10, *p* < .001).

The Kaplan–Meier curve for time to the composite outcome by population group (epilepsy vs. controls) is shown in Figure 2. The log‐rank test demonstrated a significant difference between groups (*p* < .001).

**FIGURE 2:**
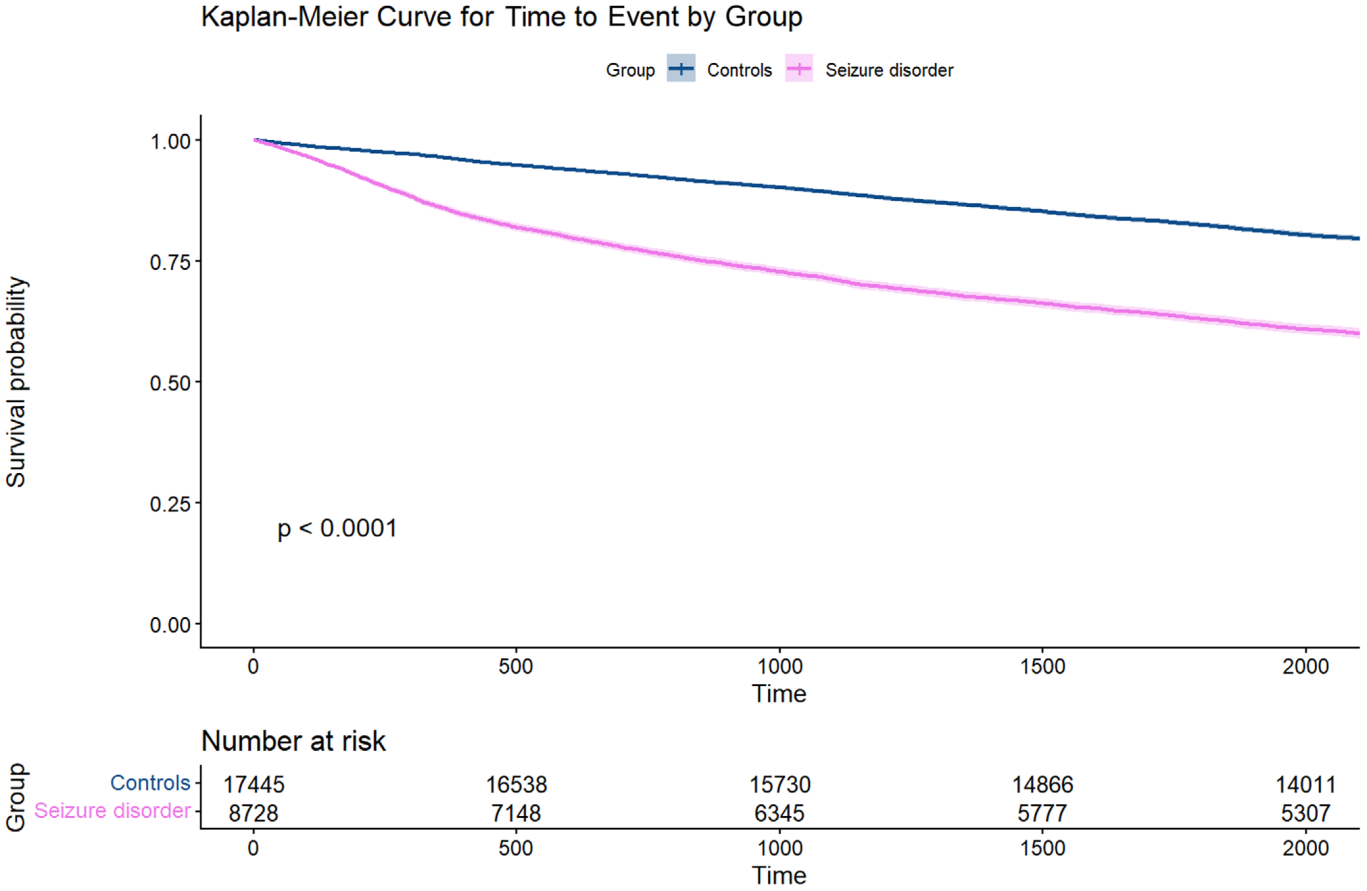
Kaplan–Meier curve for time (expressed in days) to composite event by population group (seizure disorder vs. controls). The curve shows the time (expressed in days) to the composite outcome for each group. The log‐rank test *p*‐value is shown, along with the number of participants at risk at selected time points.

### 3.3 Difference in risk between EMASMs and NEMASMs

To address the second question, a Cox regression analysis was performed on a subgroup of 1758 individuals with a seizure diagnosis and at least six consecutive months of an ASM prescription. Within this subgroup, 656 events were recorded. Increasing age remained significantly associated with a higher hazard of the outcome (HR per year = 1.05, 95% CI = 1.05–1.06, *p* < .001). Sex was not significantly associated with the hazard (female vs. male: HR = .95, 95% CI = .82–1.12, *p* = .55). Baseline cardiovascular drug use was not significantly associated with the outcome, either for one drug (HR = .91, 95% CI = .68–1.21, *p* = .52) or for more than two drugs (HR = 1.13, 95% CI = .90–1.43, *p* = .30). Use of EMASMs was associated with a nonsignificant increase in hazard compared to NEMASMs (HR = 1.14, 95% CI = .97–1.34, *p* = .12). Kaplan–Meier curves for time to the composite outcome by ASM category (EMASMs vs. NEMASMs) and treatment status (active vs. nonactive) are shown in Figure 3. The log‐rank test showed a significant difference between active and nonactive treatment (*p* < .001), but not between ASM categories (*p* = .98). Most participants initially prescribed EMASMs switched to NEMASMs during follow‐up (84.7%). In contrast, approximately half of those initially treated with NEMASMs stayed in the same ASM class (53.8%; Figure 4).

**FIGURE 3:**
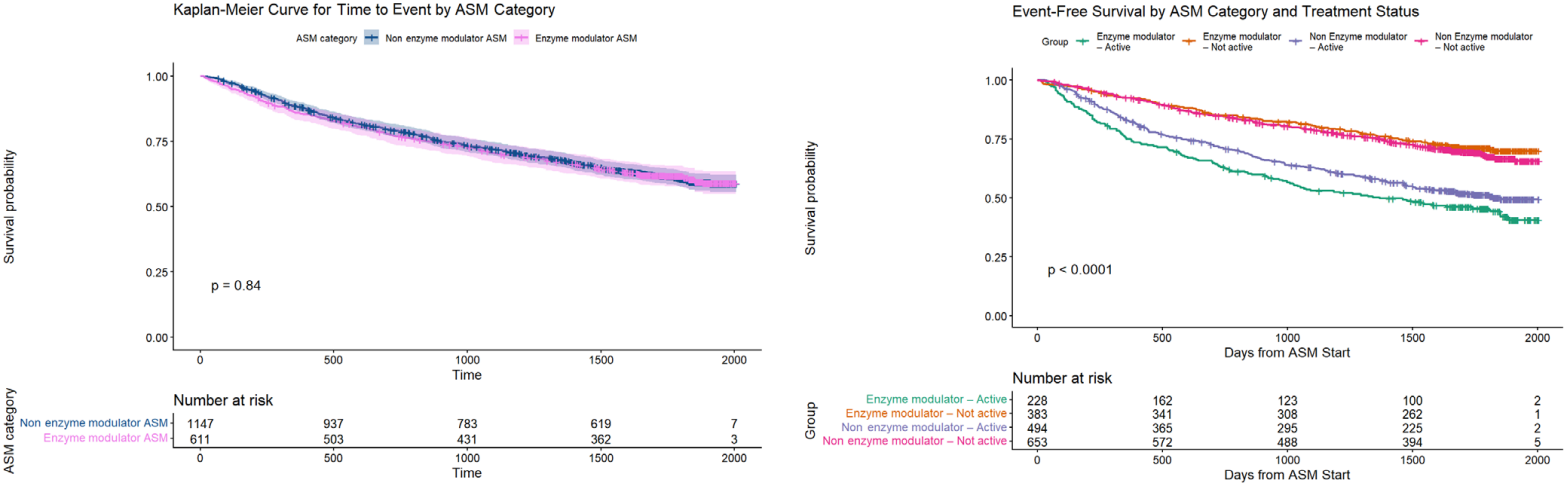
Kaplan–Meier curve for time (expressed in days) to composite event by antiseizure medication (ASM) category and treatment status. The curve shows time (expressed in days) to the composite outcome by ASM category (non‐enzyme modulating vs. enzyme modulating) and treatment status: active (prescription interval included the event date or ended within 30 days prior) versus inactive (prescription interval ended more than 30 days before the event). The log‐rank test *p*‐value is shown, along with the number of participants at risk at selected time points.

**FIGURE 4:**
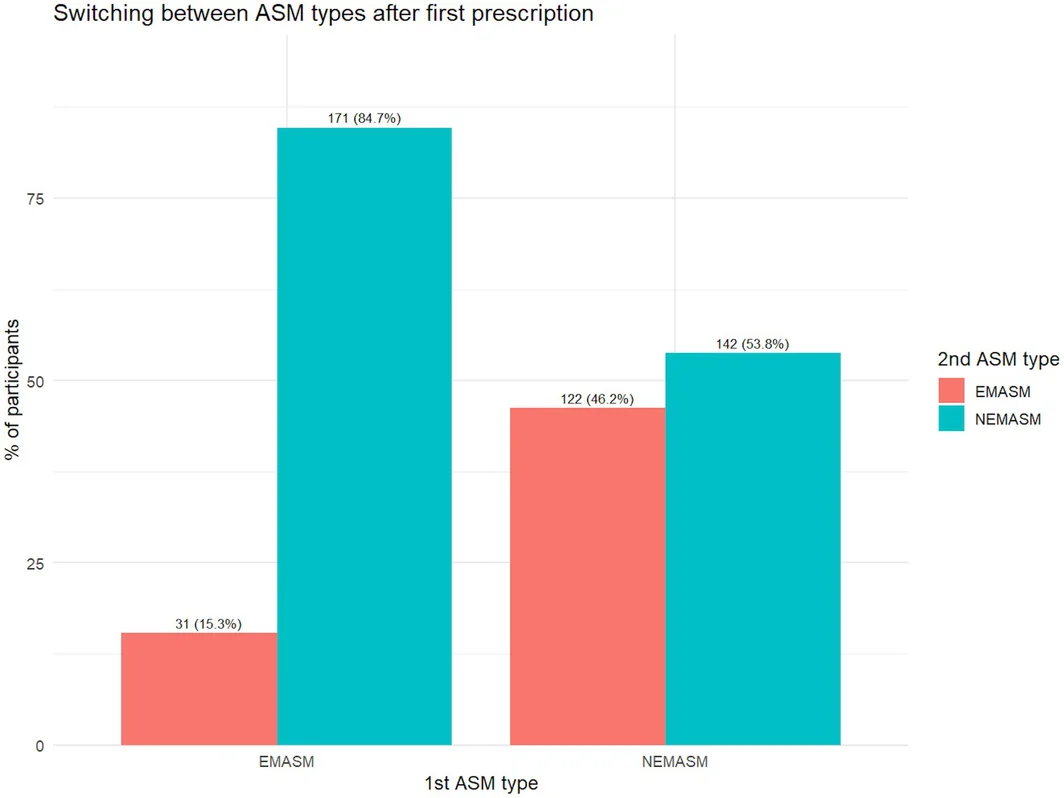
Switching of antiseizure medication (ASM) category during follow‐up, after the first ASM prescription. Distribution of ASM class during follow‐up stratified by initial ASM prescription is shown, showing the proportions of participants who remained on or switched between enzyme‐modulating antiseizure medication (EMASM) and non‐enzyme‐modulating antiseizure medication (NEMASM) classes.

### 3.4 Sensitivity analyses

A Cox regression analysis was performed in a subgroup of 2457 people with at least six consecutive months of an ASM prescription following diagnosis, regardless of indication. Eight hundred fifty‐nine events were recorded. Age did not significantly influence the hazard (HR per year = 1.00, 95% CI = 1.00–1.00, *p* < .001). Similarly, sex and baseline cardiovascular medication use were not significantly associated with the hazard (female vs. male: HR = .94, 95% CI = .82–1.08, *p* = .37; one baseline cardiovascular drug: HR = .98, 95% CI = .76–1.26, *p* = .87; more than two drugs: HR = 1.13, 95% CI = .92–1.39, *p* = .24). The use of EMASM was associated with a nonsignificant increase in hazard compared with non‐enzyme‐modulating agents (HR = 1.12, 95% CI = .97–1.30, *p* = .12).

A second subgroup analysis included 1465 individuals with a seizure diagnosis and at least six consecutive months of an ASM prescription, all of whom remained in the same category despite switching compounds. Five hundred forty‐nine events were recorded. The use of EMASMs was significantly associated with a higher hazard (HR = 1.29, 95% CI = 1.08–1.54, *p* = .01), whereas other cofactors were not (age: HR per year = 1.00, 95% CI = 1.00–1.00, *p* < .001; sex female vs. male: HR = .92, 95% CI = .78–1.10, *p* = .35; one baseline cardiovascular drug: HR = .92, 95% CI = .66–1.27, *p* = .60; more than two drugs: HR = 1.23, 95% CI = .94–1.60, *p* = .13).

In a time‐dependent analysis, we compared 1833 NEMASM users with a seizure diagnosis versus 1133 EMASM users, including 52 cases with combination therapy. One thousand two hundred eighty‐four composite events were observed during the 5‐year follow‐up. Current exposure to EMASMs, including in combination therapy, was associated with a lower risk of the composite outcome compared with NEMASMs (HR = .80, 95% CI = .70–.92, *p* = .001). Past cumulative exposure to EMASMs was associated with an increased risk (HR = 1.41 per year of exposure, 95% CI = 1.30–1.54, *p* < .001). Age was associated with higher risk (age: HR per year = 1.03, 95% CI = 1.03–1.04, *p* < .001), whereas sex and baseline cardiovascular drug use were not significantly associated with composite outcome (sex female vs. male: HR = .98, 95% CI = .88–1.09, *p* = .71; one baseline cardiovascular drug: HR = 1.11, 95% CI = .94–1.31, *p* = .20; more than two drugs: HR = 1.08, 95% CI .94–1.24, *p* = .26). Time‐varying model results with and without adjustment for cumulative EMASM exposure are summarized in Table 2.

**TABLE 2:** Time‐varying Cox regression analyses: Association between antiseizure medication category (enzyme‐modulating vs. non‐enzyme‐modulating, including combination) and risk of a composite cardiovascular outcome.

Characteristic	Model 1: Binary exposure (time‐varying)			Model 2: Binary exposure (time‐varying), accounting for cumulative exposure		
	HR	95% CI	*p*	HR	95% CI	*p*
Age	1.04	1.03–1.04	<.001	1.04	1.03–1.04	<.001
Sex						
Male	—	—		—	—	
Female	.98	.88–1.10	.7	.98	.88–1.09	.7
Cardiovascular drug prescription at baseline						
None	Reference	—	—	Reference	—	
One	1.10	.93–1.29	.3	1.11	.94–1.31	.2
More than one	1.06	.93–1.22	.4	1.08	.94–1.24	.3
Binary exposure (time‐varying)						
NEMASM	Reference	—	—			
EMASM, including in combination	1.09	.98–1.22	.12			
Binary exposure (time‐varying), accounting for cumulative exposure						
NEMASM				Reference	—	—
EMASM, including in combination				.80	.70–.92	.002
Cumulative exposure to EMASM (per 1‐year increase)				1.41	1.30–1.54	<.001

*Note*: HRs and 95% CIs were estimated using time‐varying Cox regression models. Model 1 includes binary ASM exposure (EMASM vs. NEMASM), whereas Model 2 additionally includes cumulative exposure to enzyme‐modifying ASM (per 1‐year increase). Models were adjusted for age, sex, and baseline cardiovascular drug use. Participants could contribute person‐time to multiple exposure categories. Reference categories were NEMASM, male sex, and no cardiovascular drug use.

Abbreviations: ASM, antiseizure medication; CI, confidence interval; EMASM, enzyme‐modulating ASM; HR, hazard ratio; NEMASM, non‐enzyme‐modulating ASM.

## 4 DISCUSSION

### 4.1 Cardiovascular risk following seizure diagnosis

People with seizures had a twofold higher risk of ischemic stroke or TIA, myocardial infarction, or death compared with matched controls, particularly within the first 2 years after diagnosis. This finding is consistent with previous evidence of increased cardiovascular morbidity and mortality in epilepsy, particularly early after diagnosis.^10^, ^11^ The underlying mechanisms are likely multifactorial, including comorbidities, lifestyle factors, and social determinants of health.^12^ The early excess risk may be driven by acute or preexisting mechanisms, such as shared ion channel‐related susceptibility to cardiac arrhythmias^3^, ^4^ or seizure‐related adrenergic cardiac dysfunction.^13^ Additionally, seizures may also represent an early manifestation of a previously unrecognized cerebrovascular disease in older individuals.^14^ The high burden of cardiovascular drug coprescription at baseline, which was associated with an increased risk of the composite outcome, underscores the role of comorbidities and frailty in people with seizure disorders.^15^, ^16^ As comorbidities were not directly coded, cardiovascular medication use served as a proxy. The increase in risk observed with its use is likely due to confounding by indication,^17^ as treated individuals inherently have a higher baseline cardiovascular risk. Risk increased with age and decreased in women, but these findings should be interpreted cautiously, as these variables were balanced between groups.

### 4.2 EMASMs and cardiovascular outcomes

Our analyses suggest that prolonged exposure to EMASMs may contribute to cardiovascular risk. Subgroup analyses of individuals who remained in the same ASM category despite switching compounds showed higher hazard rates with EMASMs than with NEMASMs. Similarly, cumulative prior EMASM exposure was associated with increased risk. These findings should be interpreted cautiously due to the reduced sample size and potential residual confounding. However, they are consistent with longer term studies showing that cardiovascular risk related to EIASMs emerges gradually and becomes more apparent with prolonged exposure.^18^ In particular, a cohort study with nearly 30 years of follow‐up demonstrated a dose–response association between prolonged EIASM exposure and cardiovascular events.^18^ UK clinical practice data also showed increased risks of stroke and myocardial infarction among EIASM users, particularly with prolonged valproate exposure.^5^

An increased risk was also observed in people receiving active ASM prescriptions at the time of the event, suggesting a possible role for drug–drug interactions. This may involve antihypertensive or anticoagulant therapies, potentially reducing treatment effectiveness. However, evidence remains limited, and current data support cautious interpretation rather than definitive conclusions.^19^, ^20^ Other mechanisms, including arrhythmogenic effects or drug toxicity, may also contribute to cardiovascular risk and mortality in epilepsy.^4^, ^21^, ^22^

Current or short‐term EMASM exposure appeared to be associated with lower or no risk, but this likely reflects residual confounding and prescribing bias. This is consistent with previous studies showing no clear association during shorter follow‐up periods.^23^, ^24^, ^25^

Previous studies have focused on individual ASMs rather than enzyme‐modulating properties. However, findings for specific ASMs remain inconsistent. For instance, studies have reported conflicting results regarding whether valproate increases or decreases cardiovascular risk.^26^, ^27^ Older EMASMs, like phenytoin and oxcarbazepine, have been associated with a higher risk of stroke or cardiovascular events.^26^, ^28^ It has also been suggested that the NEMASMs lamotrigine and levetiracetam are associated with a lower risk of premature mortality compared with carbamazepine.^26^, ^27^, ^29^ We avoided individual ASM categorization to preserve sample size and statistical power. Overall, heterogeneity in study design, follow‐up duration, ASM exposure definitions, and outcome measures limits direct comparisons across studies and precludes firm conclusions regarding the cardiovascular effects of EMASMs.

### 4.3 Strengths and limitations

This study has several methodological strengths, including the use of nationwide health insurance data with clinically validated coding for epilepsy and cardiovascular outcomes,^30^, ^31^, ^32^ enhancing generalizability.

Analyses were further strengthened by identifying incident cases with a 2‐year washout period, restricting outcomes to ischemic events, and using a composite endpoint to avoid informative censoring. Additionally, a rigorous ASM categorization was applied, focusing on the first prescribed ASM with a clinically meaningful treatment duration, to capture sustained exposure and allow sufficient time for potential biological effects. This selection reduced the number of participants in the final analysis, potentially limiting statistical power. Sensitivity analyses addressed potential exposure misclassification by modeling ASM use as a time‐varying covariate, adjusting for cumulative prior exposure to EMASMs. The analysis was also restricted to individuals who remained within the same ASM category despite switching compounds.

Limitations include reliance on ICD codes and prescription claims, lack of information on ASM indication, epilepsy characteristics, lifestyle factors, biological cardiovascular risk markers, comorbidities, and cause of death. This lack of information may have resulted in residual confounding, which may also have influenced the results. Using baseline cardiovascular medication as a proxy for disease burden would only partially address this. Epilepsy‐related mortality may have contributed to the higher risk of the composite outcome.^33^ This includes factors such as suicide, sudden unexpected death in epilepsy, or seizure‐related injuries. Additionally, an earlier Dutch cohort study reported excess mortality in the first 2 years following epilepsy diagnosis, which was partly attributed to epilepsy‐related causes.^34^ This mortality likely contributed to the composite outcomes observed in our cohort.

It was also not possible to assess medication adherence, combination therapy, and confounding by indication. However, ASM prescription patterns supported diagnostic validity, with analgesic ASMs predominating in individuals without seizure disorder codes and seizure‐specific ASM claims clustering around diagnosis in those with seizure disorder codes (Figure 1A,B). The requirement for sustained ASM exposure may have led to an underestimation of early events, likely due to cardiac conduction disorders.

## 5 CONCLUSIONS

People with epilepsy have an increased cardiovascular risk, particularly early after diagnosis. Prolonged cumulative exposure to EMASMs may further contribute to this risk, but these findings should be interpreted with caution.

## AUTHOR CONTRIBUTIONS

**Paola Vassallo:** Conceptualization; formal analysis and figures; writing—original draft preparation. **Mark R. Keezer:** Supervision; writing—review and editing. **Nathan A. Shlobin:** Conceptualization; review; writing—review and editing. **Roland D. Thijs:** Conceptualization; supervision; writing—review and editing. **Ley Sander:** Conceptualization; supervision; formal analysis; writing—review and editing. All authors have read and agreed to the final version of the manuscript.

## CONFLICT OF INTEREST STATEMENT

P.V. and N.A.S. have no conflicts of interest to report. M.R.K. reports unrestricted educational grants from Jazz Pharmaceuticals and Paladin. R.D.T. has received personal compensation for serving on advisory boards or speaker's bureaus for Xenon, Theravance, LivAssured, Novartis, Esai, Angelini Pharma, and UCB Pharma, as well as grants from New Life Wearables. L.S. or his department has received grants from Angelini Pharma and UCB Pharma. He has received personal compensation for consulting, serving on advisory boards, and speaking engagements from Sun Pharma, SK Life Sciences, and Angelini Pharma.

## CONSENT

We used anonymized health insurance claims data, which does not require individual informed consent.

## ETHICAL PUBLICATION STATEMENT

We confirm that we have read the Journal's position on issues involved in ethical publication and affirm that this report is consistent with those guidelines.

## Supporting information


TABLE S1

## Data Availability

The data that support the findings of this study are available on request from the corresponding author. The data are not publicly available due to privacy or ethical restrictions.
