# Supplementary material for: Cardiovascular events and mortality in adults with late onset seizures: Focus on enzyme‐modulating antiseizure medications
Source: Epilepsia. 2026 Jul 18;67(10):5243–54. doi: 10.1002/epi.70389 (PMC13675773; doi:10.1002/epi.70389)
Supplement: Supplementary file 1 [file EPI-67-5243-s001.docx]

**Supplementary Materials**

**Cardiovascular events and mortality in adults with late-onset seizures: Focus on Enzyme-modulating Antiseizure Medications.**

Paola Vassallo^1,2,3^, Nathan A. Shlobin^2,3,4^, Mark R. Keezer^5,6,7^, Roland D. Thijs^1,2,3^*, Josemir W. Sander^1,2,8^*

**Table S1** Delay between first antiseizure medication (ASM) prescription and seizure disorder diagnosis.

**Table S1 Legend** Delay (in days) between antiseizure medication (ASM) prescription and seizure disorder diagnosis. Negative values indicate prescriptions issued before the diagnosis code was recorded; positive values indicate prescriptions issued after. Mean and median values are reported for each antiseizure medication (ASM), along with interquartile range, minimum and maximum delay and total number of first antiseizure medication (ASM) prescriptions.

| **ASM** | **Mean delay** | **Median delay** | **IQR delay** | **Minimum delay** | **Maximum delay** | **n** |
| --- | --- | --- | --- | --- | --- | --- |
| Levetiracetam | 2 | 3 | 29 | -721 | 2050 | 2495 |
| Valproic Acid | -151 | 0 | 417 | -728 | 1730 | 1984 |
| Pregabalin | 79 | -167.5 | 896.5 | -702 | 2071 | 1218 |
| Carbamazepine | -329 | -419 | 437 | -722 | 1999 | 752 |
| Gabapentin | -14 | -316.5 | 794.5 | -718 | 2009 | 423 |
| Phenytoin | -292 | -379 | 525.5 | -714 | 1614 | 353 |
| Lamotrigine | -285 | -409 | 538 | -720 | 2108 | 307 |
| Clonazepam | -172 | -218 | 474 | -701 | 1684 | 293 |
| Phenobarbital | -425 | -452 | 176.5 | -706 | 1161 | 110 |
| Topiramate | -123 | -367 | 533 | -720 | 2051 | 84 |
| Oxcarbazepine | -362 | -394 | 223 | -693 | 169 | 68 |
| Primidone | -344 | -407 | 220.5 | -660 | 206 | 25 |
| Lacosamide | -271 | -351 | 485.25 | -620 | 48 | 15 |
| Ethosuximide | -452 | -446 | 183.5 | -650 | -53 | 7 |
| Vigabatrin | -512 | -525 | 230.75 | -663 | -357 | 6 |
| Retigabine | -469 | -469 | 0 | -469 | -469 | 1 |
